# Human immunodeficiency virus in chronic limb-threatening ischaemia: Risk factors, management, and outcomes: A South African retrospective cohort study

**DOI:** 10.4102/jcmsa.v4i1.321

**Published:** 2026-03-27

**Authors:** Craig Corbett, Helene Louwrens, Tonya M. Esterhuizen, Daniel C. Germishuys, Reinhard Hayes

**Affiliations:** 1 Division of Epidemiology and Biostatistics, Faculty of Medicine and Health Sciences, Stellenbosch University, Cape Town, South Africa; 2 Division of Surgery, Faculty of Medicine and Health Sciences, Stellenbosch University, Cape Town, South Africa; 3 Faculty of Medicine and Health Sciences, Stellenbosch University, Cape Town, South Africa

**Keywords:** HIV, chronic limb-threatening ischaemia, prevalence, risk factors, outcomes

## Abstract

**Background:**

People living with human immunodeficiency virus (PLHIV) have an increased risk of chronic limb-threatening ischaemia (CLTI), yet data from sub-Saharan Africa in the modern antiretroviral era remain limited. We estimated the human immunodeficiency virus (HIV) prevalence in patients admitted with index CLTI and compared risk factors and outcomes with those of their seronegative counterparts.

**Methods:**

Retrospective study of adults admitted with CLTI to Tygerberg Hospital (2016–2022). Patients identified via discharge summaries were corroborated with medical records. Clinical and HIV status data were extracted from medical and laboratory records.

**Results:**

Among 1205 patients admitted with index CLTI, the estimated prevalence of HIV was 3.5% (95% CI: 2.5% – 4.7%), with 588 confirmed HIV-negative (48.8%) and 575 with unknown HIV status (47.7%). The PLHIV cohort was 69% male compared to 64% in the HIV-negative group (*p* = 0.511). The mean age of PLHIV with CLTI was 51 years (± 9 years) compared to 62 years (± 11 years) (*p* < 0.001). People living with human immunodeficiency virus were less than half as likely to undergo endovascular therapy as their definitive management although not statistically significant (7.1% vs 17.8%; odds ratios: 0.49, 95% CI: 0.13–1.93, *p* = 0.305). A similar proportion of PLHIV and HIV-negative patients underwent open revascularisation (23.8% vs 22.8%, *p* = 0.652). Surgical management had similar rates of successful outcomes (defined as below ankle amputation or better, 90% vs 95.5%, *p* = 0.433). We found no difference in in-hospital mortality rates.

**Conclusion:**

People living with HIV were younger and had fewer risk factors for CLTI than their HIV-negative counterparts. In-hospital revascularisation outcomes, complication rates and mortality were similar.

**Contribution:**

Further study is needed regarding the district hospital HIV burden and long-term outcomes in these patients.

## Introduction

Human immunodeficiency virus (HIV) infection is an established risk factor for vascular disease.^1^ Chronic limb-threatening ischaemia (CLTI) signifies the severe phase of the peripheral artery disease (PAD) spectrum and is associated with major morbidity, limb loss and mortality.^2^ Chronic limb-threatening ischaemia is defined as ischaemic rest pain, tissue loss or gangrene of the lower extremities.^2^ In 2015, over 230 million adults worldwide were estimated to have PAD, 72.9% of whom were in low- and middle-income countries,^3^ with prevalence still increasing globally.^4^ South Africa, with the greatest absolute number of people living with human immunodeficiency virus (PLHIV) in the world,^5^ faces a significant burden of PAD.^6^ The interplay of HIV and traditional cardiometabolic risk factors is complex, particularly so with the transformation of HIV into a chronic disease as a result of improved roll-out of antiretroviral treatment (ART): ART exposure in PLHIV has improved from 26% in 2010 to 74% in 2021.^5,7^ Current data on PLHIV presenting with CLTI, especially in the ART era, remain limited.^1^

There is increasing evidence that PLHIV develop vascular disease at a younger age with fewer traditional cardiovascular risk factors than HIV-negative individuals do.^8,9,10^ This fact has been corroborated by African studies.^11,12^

The optimal management of CLTI in PLHIV remains uncertain. Some studies found PLHIV with CLTI were more likely to undergo lower extremity amputation compared to HIV-seronegative patients and had worse surgical outcomes, with higher rates of bypass failure and mortality.^13,14^ Similarly, South African data, prior to modern ART, showed poor limb-salvage rates for occlusive disease (31.6%^15^ and 36.1%^16^), with subsequent longer hospital admissions and amputation rates.^15,16^ It is important to note that these studies were limited by small sample sizes and conducted prior to the modern ART era. Consequently, drawing definitive conclusions regarding the efficacy of modern endovascular and surgical interventions in this population remains challenging. Conversely, others found no worse outcomes with endovascular revascularisation^8^ and no increased risk of amputation or mortality after surgical revascularisation.^8,17^

Our study focused on CLTI in the South African context, a population with a high burden of HIV and an increasing burden of non-communicable diseases. Our primary aim was to establish the period prevalence of HIV among patients with index CLTI at Tygerberg Hospital, a tertiary care facility in Cape Town, South Africa. Secondary aims included describing the HIV-specific characteristics of PLHIV with CLTI, as well as comparing the age distribution, cardiovascular risk factors, comorbidities and management outcomes of PLHIV presenting with CLTI to their HIV-negative counterparts.

## Research methods and design

### Study design and setting

We conducted a retrospective review of electronic medical records (EMRs) of adults admitted to the Vascular Surgery Department of Tygerberg Hospital from 01 January 2016 to 31 December 2022. Tygerberg Hospital is a public tertiary healthcare institution serving a large drainage area with both urban and rural South Africans and foreign nationals of various ethnicities.^18^

### Participant identification

**FIGURE 1:**
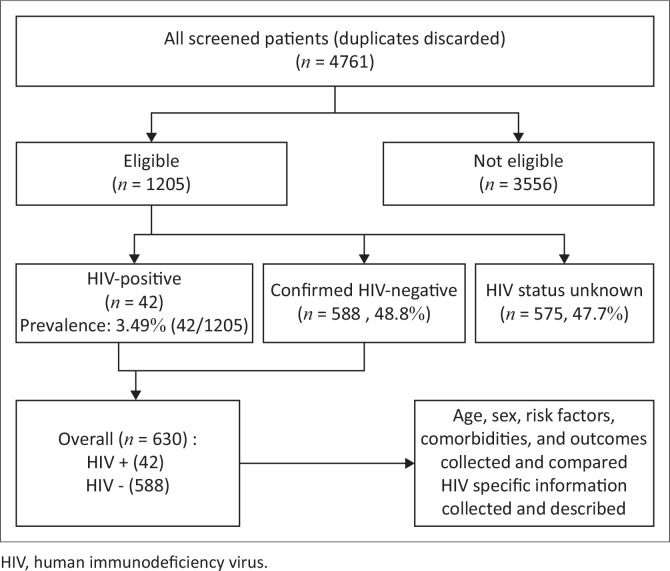
Flow diagram illustrating patient selection and data collected.

Adults ≥ 18 years with index CLTI (ischaemic rest pain, tissue loss or gangrene of the lower extremities for ≥ 2 weeks) were included.^2^ Patients were identified from the Vascular Surgery Department discharge database, and after being screened for eligibility, the diagnosis of CLTI was established by further analysing clinical information retrieved from EMRs (see Figure 1). Thereafter, the HIV status of all eligible patients was determined from laboratory data on the National Health Laboratory Service database.

Patients were excluded if admitted for CLTI before January 2016, had major lower limb amputation prior to admission, had alternative diagnoses (e.g. acute limb ischaemia, trauma, cancer-related vascular disease, upper limb CLTI and aneurysms) or lacked available EMRs.

### Outcomes and data collected

Detailed clinical data were collected from EMRs and laboratory results for all eligible participants and entered into a password-protected standardised Excel spreadsheet. Data on demographics, cardiovascular risk factors, current infections (including pulmonary tuberculosis), established atherosclerotic disease and major comorbidities were extracted.

The virologic, immunologic and ART statuses were collected for each PLHIV with CLTI. We attributed a value of 50 ribonucleic acid (RNA) copies/mL to undetectable viral loads, with suppression defined as < 200 RNA copies/mL. Viral loads and cluster of differentiation 4+ (CD4+) counts closest to presentation were recorded. Antiretroviral treatment timing was determined from pharmacy refill records.

Primary management strategies were classified as medical management (MM), endovascular therapy (ET), open surgical revascularisation (OSR), primary amputation or down-referral to a secondary facility. Medical management (risk factor reduction and secondary prevention) was assumed to accompany surgical management. Diagnostic digital subtraction angiography without any angioplasty or stenting performed was not included in ET; similarly, if performed in a hybrid procedure with open surgery (revascularisation or amputation), then this was grouped under the respective surgery. Patients declining surgery received MM and were classified as such. The level of revascularisation (aorta-iliac, femoral-popliteal, distal [trifurcation and below]), outcomes, intensive care unit (ICU) admission and in-hospital complications were recorded. A successful outcome was defined as minor amputation (below the ankle) or better, whereas an unsuccessful outcome was defined as major amputation or ongoing rest pain after revascularisation. Outcomes and complications were recorded within the index hospital admission.

The hospital stay duration was determined from the initial admission date to the date of discharge or death.

### Statistical considerations

Human immunodeficiency virus prevalence was expressed as a proportion, using the entire eligible sample, and reported with 95% confidence intervals. Human immunodeficiency virus-related information for the PLHIV with CLTI was described by using frequencies and percentages. We compared the age, sex, risk factors and management outcomes of patients with and without HIV. Continuous variables were categorised into clinically meaningful groups. Categorical variables were compared with the Pearson χ^2^ test or Fisher’s exact test, and numerical variables were compared with an independent samples *t*-test or a Mann–Whitney test, depending on the distribution of the data. Multivariable logistic regression models were used to estimate adjusted odds ratios (OR) and 95% confidence intervals for factors associated with the outcome of HIV-positive compared to HIV-negative, incorporating relevant covariates that reached a significance level of 0.1 in unadjusted analyses. Statistical significance was defined as a *p*-value of < 0.05. Data were analysed by using IBM SPSS Statistics for Windows, version 29 (IBM Corp, Armonk, NY).

### Ethical considerations

Ethical approval was obtained from the Human Research Ethics Committee of the Faculty of Medicine and Health Sciences of Stellenbosch University (S23/11/284), and permission from the relevant authorities was secured.

## Results

### Demographics

Between 2016 and 2022, a total of 1205 patients were admitted to Tygerberg Hospital with index CLTI. Of these, 42 (3.5%) were HIV-positive, 588 (48.8%) were HIV-negative and 575 (47.7%) had an unknown HIV status, yielding an HIV prevalence of 3.5% (95% CI: 2.5% – 4.7%) (Figure 1). If we consider the prevalence of 5.6% of adults > 50 years in the Western Cape,^19^ an additional 32 PLHIV in the unknown group (*n* = 575) could be assumed.

The mean age of PLHIV with CLTI was 51 years (± 9 years) compared to 62 years (± 11 years) in the HIV-negative group (*p* < 0.001). The sex of patients did not differ significantly. The baseline characteristics are presented in Table 1.

**TABLE 1:** Characteristics of human immunodeficiency virus-positive compared to those of human immunodeficiency virus-negative patients admitted with chronic limb-threatening ischaemia at Tygerberg Hospital between 01 January 2016 and 31 December 2022.

Characteristics of patients	HIV+ (*n* = 42)					HIV− (*n* = 588)					Overall (*n* = 630)				Crude *p*-value	Adjusted OR	95% CI	Adjusted *p*-value
	Median	IQR	*n*	%	s.d.	Median	IQR	*n*	%	s.d.	Median	IQR	*n*	%				
Demographics	-	-	-	-	-	-	-	-	-	-	-	-	-	-	-	-	-	-
Age (years)	51	44–57	-	-	-	63	55–69	-	-	-	62	55–69	-	-	< 0.001	0.94	0.90–0.97	< 0.001
Male sex at birth	-	-	23	54.8	-	-	-	367	62.4	-	-	-	-	-	0.324	-	-	-
Medical comorbidities	-	-	-	-	-	-	-	-	-	-	-	-	-	-	-	-	-	-
Hypertension	-	-	20	47.6	-	-	-	461	78.4	-	-	-	481	76.3	< 0.001	0.75	0.34–1.63	0.466
Diabetes mellitus	-	-	10	23.8	-	-	-	282	48.0	-	-	-	292	46.3	0.002	0.60	0.27–1.37	0.227
*Mean HbA1c* †	-	-	-	10.4	2.83	-	-	-	9.2	2.27	-	-	-	-	0.102	-	-	-
Dyslipidaemia	-	-	13	31.0	-	-	-	311	52.9	-	-	-	324	51.4	0.006	0.76	0.35–1.61	0.467
*Mean tChol* ‡	-	-	-	4.82	1.37	-	-	-	5.00	1.77	-	-	-	-	0.705	-	-	-
Previous stroke or TIA	-	-	1	2.4	-	-	-	87	14.8	-	-	-	88	14.0	0.025	0.20	0.03–1.50	0.116
IHD	-	-	1	2.4	-	-	-	97	16.5	-	-	-	98	15.6	0.015	0.23	0.03–1.74	0.153
Other cardiac pathology§	-	-	3	7.1	-	-	-	45	7.7	-	-	-	48	7.6	0.904	-	-	-
Other medical co-morbidities¶	-	-	3	7.1	-	-	-	164	27.9	-	-	-	167	26.5	0.003	0.30	0.09–1.03	0.055
Current infection	-	-	7	16.7	-	-	-	77	13.1	-	-	-	84	13.3	0.511	-	-	-
Pulmonary TB	-	-	3	7.1	-	-	-	16	2.7	-	-	-	19	3.0	0.105	1.03	0.25–3.06	0.971
Other infections††	-	-	4	9.5	-	-	-	61	10.4	-	-	-	65	10.3	0.861	-	-	-
Health-related behaviours	-	-	-	-	-	-	-	-	-	-	-	-	-	-	-	-	-	-
Current smoker	-	-	22	52.4	-	-	-	369	62.8	-	-	-	391	62.1	0.181	-	-	-
Ex-chronic smoker	-	-	5	11.9	-	-	-	71	12.1	-	-	-	76	12.1	0.974	-	-	-
Chronic alcohol use	-	-	2	4.8	-	-	-	17	2.9	-	-	-	19	3.0	0.493	-	-	-
Substance use‡‡	-	-	5	11.9	-	-	-	15	2.6	-	-	-	20	3.2	< 0.001	0.88	0.25–3.06	0.838

HbA1c%, Haemoglobin A1C percentage; tChol, total cholesterol; TIA, transient ischaemic attack; IHD, ischaemic heart disease; TB, tuberculosis; CKD, chronic kidney disease; COPD, chronic obstructive pulmonary disease; IQR, interquartile range; s.d., standard deviation; OR, odds ratio; HIV, human immunodeficiency virus; CI, confidence interval; COVID-19, coronavirus disease 2019.

† , In those with diabetes mellitus (HIV+, *n* = 10 & HIV−, *n* = 282).

‡ , In those with hypercholesterolaemia (HIV+, *n* = 13 & HIV−, *n* = 311).

§ , Congestive failure (*n* = 28), cardiomyopathy (*n* = 22) and primary valvular pathology (*n* = 7). Note: Total does not equal 48, because patients could be having more than one cardiac pathology.

¶ , COPD (*n* = 105), arrhythmia (*n* = 21), CKD (*n* = 20); neurological (*n* = 16); haematological (*n* = 4); rheumatological (*n* = 9). Note: Total does not equal 167, because patients could be having more than one comorbidity.

†† , Localised soft tissue infection (*n* = 52), local and/or systemic sepsis (*n* = 4), COVID-19 (*n* = 4); current syphilis (*n* = 3); other (*n* = 2).

‡‡ , Consisted of methamphetamines, mandrax (methaqualone), cannabis or a combination of illicit substances.

### Risk factors and comorbidities

We compared PLHIV with CLTI (*n* = 42) to confirmed HIV-negative patients with CLTI (*n* = 588). Hypertension (47.6% vs 78.4%, *p* < 0.001), diabetes (23.8% vs 48%, *p* = 0.002) and hypercholesterolaemia (31.0% vs 52.9%, *p* = 0.006) were less prevalent in PLHIV on univariate analysis. The mean HbA1c% did not differ significantly among diabetics in the two groups. Health-related behavioural risk factors showed no difference in smoking or alcohol use, but illicit substance use was more frequent in PLHIV (11.96% vs 2.6%, *p* < 0.001). Multivariable logistic regression models, adjusted for age, showed that traditional and behavioural risk factors that were different between the groups in univariate analysis no longer remained statistically significantly different (Table 1). Age remained a significant factor, and with each year in age, there was 6% less chance of being HIV-positive (OR: 0.94, 95% CI: 0.90–0.97).

Established atherosclerotic disease, specifically ischaemic heart disease (IHD) (2.4% vs 16.5%, *p* = 0.015) and stroke (2.4% vs 14.8%, *p* = 0.025), was less prevalent in PLHIV on univariate analysis but not after adjustment for age. Other comorbidities, such as chronic kidney disease, chronic obstructive pulmonary disease, atrial fibrillation and other neurological and rheumatological disorders, were borderline non-significantly more common in patients without HIV (OR: 0.3, 95% CI: 0.09–1.03, *p* = 0.055).

### Description of people living with human immunodeficiency virus with chronic limb-threatening ischaemia

Of the 42 PLHIV admitted with CLTI in this study period, a quarter were newly diagnosed with HIV (Table 2). Nearly half were using ART (45.2%), and of those on ART at the time of admission (*n* = 19), 63.2% were virally suppressed.

**TABLE 2:** Virological, immunological and antiretroviral statuses of people living with human immunodeficiency virus admitted with chronic limb-threatening ischaemia at Tygerberg Hospital between 01 January 2016 and 31 December 2022.

Characteristic	Overall values					
	Median	IQR	*n*	%	Mean	s.d.
**Virological status (*n* = 36)**						
Virally suppressed	-	-	18	50.0	-	-
Log viral load	2.18	1.70–3.69	-	-	-	-
**Immunological status (*n* = 38)**						
Absolute CD4 count	373.00	185.00–537.00	-	-	-	-
CD4%	-	-	-	-	21.14	9.82
CD4 nadir	353.00	166.00–446.00	-	-	-	-
**ART status (*n* = 42)**						
On ART	-	-	19	45.2	-	-
On ART > 6 months	-	-	17	40.5	-	-
Defaulted prior to admission	-	-	9	21.4	-	-
ART naïve	-	-	14	33.3	-	-
New diagnosis	-	-	11	26.2	-	-

CD4, cluster of differentiation 4; ART, antiretroviral therapy; IQR, interquartile range; s.d., standard deviation.

In those exposed to ART prior to admission (*n* = 28, 66.7%), 2 (7%) had been started within the previous 6 months, 9 (32%) had defaulted treatment, and 17 (61%) had consistent ART use for more than 6 months. There were no significant differences in other risk factors for CLTI when comparing subgroups by CD4+ (< / ≥ 200 cells/μL), viral suppression or ART status, except that virally suppressed patients were more likely smokers (72.2 vs 38.9%, *p* = 0.044).

Interestingly, no patients with CD4+ counts < 200 cells/µL^3^ underwent ET or OSR (*n* = 9). Of the 13 patients who underwent revascularisation, the overall success rate was 84.6% (*n* = 11) (Table 3). Neither patient with unsuccessful revascularisation was virally suppressed.

**TABLE 3:** Treatment received and surgical success rates of patients admitted with chronic limb-threatening ischaemia at Tygerberg Hospital between 01 January 2016 and 31 December 2022.

Characteristic	HIV+		HIV−†		Overall		*p*-value‡‡
	*n*	%	*n*	%	*n*	%	
Treatment received	42	-	584	-	626	-	0.203
Medical treatment‡	7	16.7	118	20.2	125	20.0	-
Endovascular therapy	3	7.1	104	17.8	107	17.1	-
Open revascularisation	10	23.8	134	22.8	144	23.0	-
Combined amputations§	22	52.4	228	39.0	250	39.9	-
Primary major amputation	10	23.8	158	26.9	168	26.7	-
Down-referral for amputation	12	28.6	70	11.9	82	13.1	-
Success	13	-	238	-	251	-	-
Endovascular therapy¶	2	66.7	95	91.3	97	90.7	0.257
Open revascularisation††	9	90.0	128	95.5	137	95.1	0.433

OR, odds ratio; HIV, human immunodeficiency virus.

† , Four HIV− patients died prior to receiving any treatment.

‡ , Medical treatment includes those initially selected for revascularisation or amputation, but who refused, leaving medical therapy as the alternative.

§ , The assumed composite outcome of amputation is a combination of primary major amputation and down-referral, assuming that all referred patients underwent amputation (and not an alternative, such as refusal or only medical therapy).

¶ , In those receiving endovascular therapy as their final procedure (HIV+, *n* = 3 & HIV−, *n* = 104).

†† , In those receiving open revascularisation as their final procedure (HIV+, *n* = 10 & HIV−, *n* = 134).

‡‡ , Pearson chi-square utilised.

### Management outcomes

Of the 626 patients who received treatment (four patients died before intervention), overall, the management received did not differ between PLHIV and HIV-negative patients (*p* = 0.203), although fewer PLHIV received (7.1% vs 17.8%). A similar proportion of PLHIV and HIV-negative patients underwent OSR (23.8% vs 22.8%), as well as primary amputation at our department (23.8% vs 26.9%) (Table 3). However, PLHIV were almost three times more likely to be down-referred to their base hospital for amputation than their HIV-negative counterparts (28.6% vs 11.9%). The anatomical level of intervention (categorised by the most proximal segment treated: aorto-iliac, femoro-popliteal or distal) was similar between the two groups for both endovascular and open procedures (Table 4).

**TABLE 4:** Highest level of revascularisation of human immunodeficiency virus-positive compared to that of human immunodeficiency virus-negative patients admitted with chronic limb-threatening ischaemia.

Level of revascularisation	HIV+ (*n* = 42)		HIV− (*n* = 588)		Overall (*n* = 630)		*p*-value‡
	*n*	%	*n*	%	*n*	%	
**Endovascular therapy**	3	7.1	104.0	17.7	107	17.0	0.724
Aorto-iliac	1	33.3	19.0	18.3	20	18.7	-
Fem-pop	1	33.3	57.0	54.8	58	54.2	-
Distal†	1	33.3	28.0	26.9	29	27.1	-
**Open revascularisation therapy**	10	23.8	134.0	22.8	144	22.9	0.256
Aorto-iliac	3	30.0	28.0	20.9	31	21.5	-
Fem-pop	6	60.0	10.3	76.9	109	75.7	-
Distal†	1	10.0	3.0	2.2	4	2.8	-

Fem-pop, femoral-popliteal; HIV, human immunodeficiency virus.

† , Distal refers to trifurcation level and below.

‡ , Pearson chi-square utilised.

Endovascular therapy had less successful outcomes in PLHIV compared to HIV-negative patients although the numbers in the HIV group were low and the difference was not statistically significant (66.7% vs 91.3%, *p* = 0.257). Open surgical revascularisation success rates did not differ significantly (90% vs 95.5%, *p* = 0.433).

### Complications, in-hospital mortality and hospital stay length

Overall, in-hospital mortality was slightly higher in PLHIV although not statistically significant (4.8% vs 1.4%, *p* = 0.088). Only one of these deaths occurred after OSR, and this was in an HIV-negative patient (Table 5). Similarly, overall complications (including wound infection, haematoma and death) were comparable. Among those who underwent revascularisation, complication rates did not differ (7.7% vs 11.3%, *p* = 0.684). We found no difference in length of hospital stay, regardless of the subgroup analysed, including living patients only, transferred or non-transferred patients or all patients.

**TABLE 5:** Mortality, complications and hospital stay length.

Characteristic	HIV+ (*n* = 42)				HIV− (*n* = 588)				Overall (*n* = 630)				*p*-value§
	Median	IQR	*n*	%	Median	IQR	*n*	%	Median	IQR	*n*	%	
In-hospital mortality	-	-	2	4.8	-	-	8	1.4	-	-	10	1.6	0.088
Post-revascularisation mortality†	-	-	0	0.0	-	-	1	0.7	-	-	1	0.7	1.000
Complications during hospital stay‡	-	-	4	9.5	-	-	38	6.5	-	-	42	6.7	0.442
ICU admission (not PACU)	-	-	1	2.4	-	-	20	3.4	-	-	21	3.3	0.722
Median length of ICU stay, days, IQR	-	*Only 1 pt*	-	-	3.0	1–5.0	-	-	3	2–5	-	-	n/a
**Hospital stay length in days**													
All patients (*n* = 630)	7.5	4–16	-	-	10.0	4–17.0	-	-	-	-	-	-	0.216¶
Only living patients (*n* = 620)	7.5	4–15	-	-	10.0	4–17.0	-	-	-	-	-	-	0.267¶
Deceased patients (*n* = 10)	8.5	1–16	-	-	10.5	6–18.5	-	-	-	-	-	-	0.711¶
Non-transferred patients (*n* = 498)	10.0	6–24	-	-	12.0	6–19	-	-	-	-	-	-	0.713¶
Transferred patients (*n* = 122)	4.0	2–10	-	-	4.0	2–8.0	-	-	-	-	-	-	0.750¶

ICU, intensive care unit; PACU, post-anaesthesia care unit; NSTEMI, non-ST elevation myocardial infarction; HIV, human immunodeficiency virus; IQR, interquartile range.

† , Of patients undergoing open surgery: HIV+ (*n* = 10) and HIV− (*n* = 134).

‡ , Consisted of wound infection (*n* = 24), lymph leak and/or haematoma (*n* = 10), NSTEMI (*n* = 1), Deep Vein Thrombosis (*n* = 2) and death (*n* = 4). Complications did not include failed grafts (as these were included in the outcomes in Table 3).

§ , Pearson chi-square utilised.

¶ , Mann-Whitney U test asymptotic (two sided).

## Discussion

Our study found a minimum HIV prevalence of 3.5% (95% CI: 2.5% – 4.7%) among index CLTI patients admitted over a 7-year period in a high HIV-burden population during the ART era. People living with human immunodeficiency virus presented more than a decade younger and were less likely to have hypertension, diabetes, hypercholesterolaemia, IHD and stroke although multivariable analysis suggested these differences were largely age related. Importantly, no significant differences were observed in endovascular or OSR success, complication rates or in-hospital mortality in PLHIV compared to their HIV-seronegative counterparts; these findings are qualified by the small number of PLHIV in the treatment subgroups. Consequently, these results should be interpreted as observational trends rather than definitive clinical outcomes, particularly regarding the comparative efficacy of surgical versus endovascular interventions.

Notably, 26% of PLHIV were newly diagnosed during their admission, and one-third were ART naïve. This finding suggests that CLTI often serves as the sentinel presentation of untreated HIV in younger patients. These findings highlight a critical opportunity for vascular units to implement routine HIV screening and initiate ART early, which may be vital in mitigating the systemic inflammation and accelerated vascular ageing that drive progression to limb-threatening ischaemia.

Our estimated prevalence is lower than the latest reported HIV prevalence of 5.6% of the adults aged 50 years and older in the Western Cape.^19^ Although our prevalence calculation is conservative, there lies a possibility that PLHIV present at an unsalvageable stage in their CLTI disease at their base hospitals and therefore undergo primary amputation without tertiary referral. Tran et al. found that symptomatic PLHIV were more likely to present with advanced disease of PAD than their seronegative counterparts.^13^ People living with human immunodeficiency virus in our study were almost three times more likely to be down-referred for amputation, which may suggest that more PLHIV are being referred in from our district hospitals than those presenting within our own drainage area.

The mechanisms promoting earlier CLTI in PLHIV remain multifactorial, likely involving cytokine-driven endothelial dysfunction and accelerated atherosclerosis that persists despite viral suppression.^20^ Our finding that PLHIV presented with a lower prevalence of traditional risk factors like hypertension and diabetes mirrors previous research,^8,13,17,21^ although our multivariable analysis suggests that this is largely a function of their younger age. This result suggests that while HIV may accelerate vascular ageing, this paucity of comorbidities did not differ when stratified by CD4+, viral load or ART status.

The high rate of amputation observed in our study confirms that CLTI remains a morbid end stage of PAD in the South African context, aligning with pre-modern ART studies by Van Marle and Paruk.^16,22^ This outcome suggests that despite advances in HIV care, many patients still present with unsalvageable disease. Furthermore, while PLHIV were less likely to undergo ET, this situation reflects international trends in which HIV-related lesions are often more complex, advanced (Rutherford ≥ 4) and less amenable to percutaneous intervention.^13^ Notably, the absence of revascularisation in patients with CD4+ counts < 200 cells/µL may suggest that severely immunocompromised PLHIV present later or with more aggressive disease, potentially precluding limb-salvage efforts.

We found no increased likelihood of worse perioperative outcomes – specifically regarding major amputation, complications or mortality – in PLHIV compared to HIV-negative patients. These findings align with recent large retrospective cohorts showing no significant difference in perioperative success among procedure-matched patients regardless of HIV status.^17^ Our success rates for OSR are consistent with international literature, including outcomes from major randomised controlled trials such as Farber et al.^23^ While pre-modern ART studies in South Africa reported lower salvage rates,^22^ contemporary data suggest that revascularisation is increasingly effective, though regional variations in disease severity and healthcare infrastructure continue to influence individual outcomes.

We recognise that the limitations of our study lie initially in the retrospective nature thereof. We were reliant on the quality of existing EMRs, and certain health determinants could not be determined retrospectively, such as socioeconomic status, obesity or healthcare access. This was a public healthcare single-centre study, and inter-hospital HIV prevalence may vary. Almost half of the cohort (47.7%) had an unknown HIV status, which we conservatively assumed to be HIV-negative, potentially underestimating HIV prevalence. People living with human immunodeficiency virus numbers were marginally less than expected, which may have limited the power to detect significant differences in certain outcomes or subgroups. We did not stratify patients on their clinical presentation or prognostication tools such as the Wound, Ischaemia and Foot Infection (WIfI) score, which may affect outcomes.^24^ We are a resource-limited setting, and hence, poor surgical candidates would not be selected for revascularisation. We further assumed that all down-referred patients underwent amputation, as revascularisation is unavailable outside tertiary facilities; however, some patients may have been deemed terminal and received palliation instead. Furthermore, as outcomes and complications were recorded only during the index hospital admission, this study does not capture long-term limb-salvage rates, late-onset complications or readmissions for recurrent symptoms, which may lead to an underestimation of the total disease burden. Down-referral may thus underestimate in-hospital mortality. Surgical decisions, such as primary ablation versus revascularisation, were assumed to rely on established patient prognostic factors, such as age, comorbidities, extent of tissue loss, sepsis, venous conduit availability, adequate target vessels, mobility and patient preferences – rather than HIV status alone. The lack of post-discharge follow-up is a significant methodological limitation, compounded by the well-documented challenges of patient retention and fragmented referral pathways within the South African public healthcare system. Consequently, while our in-hospital outcomes are robust, the reliability of our conclusions regarding long-term limb salvage and mortality remains restricted, as we cannot account for late-stage complications or deaths occurring after discharge. We did not track patients after discharge from the index intervention, preventing identification of post-discharge complications or surgical failures.

Future research could focus on revascularisation outcomes in age-matched prospective studies, addressing aspects such as socioeconomic factors, WIfI scores and post-discharge follow-up. Furthermore, the number of down-referred PLHIV prompts consideration of the burden of HIV with CLTI in district hospitals, including whether more PLHIV are undergoing primary amputation than HIV-negative individuals, without referral for revascularisation work-up.

## Conclusion

In conclusion, we found a minimum HIV prevalence of 3.5% in patients admitted for index CLTI to Tygerberg Hospital from 2016 to 2022. People living with human immunodeficiency virus were 12 years younger, with fewer risk factors than their HIV-negative counterparts, yet revascularisation outcomes, complication rates and in-hospital mortality were similar. Chronic limb-threatening ischaemia remains a significant consequence of the double-burden of HIV and non-communicable diseases in resource-limited settings. Our results suggest that revascularisation may be a viable strategy in managing these patients; such observational trends, if confirmed in larger cohorts, could support more equitable treatment approaches. Early diagnosis, timely referral and prevention strategies, such as risk factor reduction and consistent ART adherence, may be essential to improving outcomes in PLHIV with CLTI. Frontline clinicians should recognise CLTI in younger PLHIV with fewer risk factors to enable early intervention. Further research is required to better understand the challenges faced by PLHIV with CLTI.
